# Purification of Polyphenols from Distiller’s Grains by Macroporous Resin and Analysis of the Polyphenolic Components

**DOI:** 10.3390/molecules24071284

**Published:** 2019-04-02

**Authors:** Xiaoyuan Wang, Shuangshuang Wang, Shasha Huang, Lihua Zhang, Zhenzhen Ge, Liping Sun, Wei Zong

**Affiliations:** 1 School of Food and Bioengineering, Zhengzhou University of Light Industry, Zhengzhou 450002, Henan, China; wangsh0521@163.com (S.W.); huangsha1514@163.com (S.H.); zhanglihua8@yahoo.com (L.Z.); gezzfood@163.com (Z.G.); sunliping78@126.com (L.S.); 2 Collaborative Innovation Center of Food Production and Safety, Zhengzhou 450002, Henan, China

**Keywords:** distiller’s grains, polyphenols, macroporous resin, purification

## Abstract

We aimed to purify polyphenols from distiller’s grain extract using macroporous resins and to identify its polyphenolic components. The influence of operational parameters on purification efficiency was investigated. The polyphenolic composition was analyzed by ultra-performance liquid chromatography tandem mass spectrometry (UPLC-MS/MS) and then quantified by UPLC-MS using authenticated standards. The results showed that the optimal purifying conditions were D101 resin with a dosage of 3 g, four hours adsorption, three hours desorption time, and 60% ethanol as the eluent, producing the highest purification rate of 51%. The purified distiller’s grain extract exhibited stronger antioxidant activity than the unpurified extracts, which was assessed using DPPH and ABTS methods (IC_50_ DPPH = 34.03 and 16.21 μg/mL, respectively; IC_50_ ABTS = 20.31 and 5.73 μg/mL, respectively). UPLC-MS results indicated that (−)-epicatechin is the major compound found in distiller’s grain extract which was quantified as 562.7 μg/g extract, followed by ferulic acid (518.2 μg/g), *p*-hydroxybenzoic acid (417.7 μg/g), caffeic acid (217.1 μg/g), syringic acid (158.0 μg/g) and quercetin (147.8 μg/g). Two compounds, vanillic acid (66.5 μg/g) and gallic acid (41.4 μg/g), were found in lower concentrations. The findings of this study suggest that purification of polyphenolic compounds from distiller’s grain by macroporous resins is feasible, providing a new and effective method for the secondary use of distiller’s grain resources.

## 1. Introduction

Distiller’s grains are the by-products of fermentation and distillation of cereal crops [1]. There are two main sources of these grains: by-product from the liquor manufacturing industry, and by-products from the fuel ethanol industry. Distiller’s grain contains many components including starch, protein, cellulose, organic acids, amino acids, vitamins, fats, liquor flavor substances, phenolic compounds, nitrogen compounds, and heterocyclic compounds [2,3]. Because of the high water content and high acidity, fresh distiller’s grains easily rot and deteriorate if they are discharged and not treated in time, causing serious environmental damage and wasting a large amount of resources. Over the past decade, the fuel-ethanol industry has experienced a phenomenal surge in growth worldwide. Global production of bioethanol was 46 billion liters in 2007, whereas in the United States alone, ethanol production was up to 39 billion liters in 2009 [4,5]. Huge amounts of liquor were also produced. According to statistics, more than 1563 Chinese scale liquor enterprises were formed in 2015, and the annual output of dry distillers’ grains reached 4 million tons. Considering the high yield of ethanol biofuels and liquor, the treatment of distiller’s grains is an unavoidable problem for large and medium-sized ethanol biofuels and liquor producing enterprises. The comprehensive use of distiller’s grains resources will contribute to the sustainable development of liquor-making enterprises.

Regarding the in-depth study of the composition of distiller’s grains, many scholars have focused on the use of distiller’s grains to produce organic fertilizers, edible fungi, protein feeds, and vinegar [6,7]. Some researchers have used distiller’s grains as raw materials for further fermentation to obtain succinic acid and xylitol. Poonam et al. applied food-grade distiller’s dried grains, garbanzo flour, and corn grits to produce extrudates and assessed the physicochemical and nutritional properties of the extrudates [8]. However, it is still not possible to fully convert the distiller’s grains into useful resources.

Many reports have been published on the general composition and variability of distiller’s grains. Liu et al. found that the average values (% dry matter) for protein, oil, ash and starch were 27.4, 11.7, 4.4 and 4.9%, respectively, on a dry matter basis [9]. Later, the same group investigated the content of both essential and nonessential amino acids, including Arg, His, Ile, Leu, Lys, Met, Phe, Thr, Trp and Val. [10]. Several research groups observed that distiller’s grains contained many kinds of trace elements essential for the human body [11,12,13]. According to the literature, the fatty acid composition of distiller’s grains oil is as follows: linoleic acid (53.96–56.53%), followed by oleic acid (25.25–27.15%) and palmitic acid (13.25–16.41%), with low levels of stearic (1.80–2.34%) and linolenic (1.15–1.40%) acids [1,14,15]. Vermont et al. evaluated the effect of processing method and corn cultivar on the anthocyanin concentration from dried distillers drains with solubles [16]. Although many studies have been published on the composition of distiller’s grains, few studies have reported the composition of polyphenols in distillers’ grains.

Polyphenols are active substances widely present in plants and performed many physiological functions. They not only have strong free radical scavenging ability, but also play an anti-oxidation role by inhibiting oxidase and complex transition metal ions [17]. Given the problems caused by the discards of wine grains and the advantages of polyphenols, the polyphenols in wine grains were extracted and purified in the present study, laying a foundation for the efficient development and use of a large number of polyphenols in white wine grains, and providing new possibilities for the secondary use of white wine grains resources.

## 2. Results and Discussion

### 2.1. Purification of Polyphenols by Macroporous Resin

#### 2.1.1. Screening of Macroporous Resin Type and Dosage

The adsorption properties of macroporous adsorbent resins are related to their surface properties, pore structure, and solubility of adsorbents. Table 1 displays the adsorption and desorption rates of six types of macroporous resins. The significant difference in adsorption and desorption efficiency among the tested resins demonstrated that the adsorption and desorption of the polyphenol from distiller’s grain by resin is selective. The highest adsorption rate was achieved by D101, HPD400 and NKA-9, whereas the lowest adsorption rate was observed in the middle polar resin-HPD750. According to the properties of the six tested resins shown in Table 1, the adsorption capacity for polyphenol is not only related to the polarity of the resin. Generally, the adsorption efficiency increased with specific surface area. Here, all the resins presented this trend except for HPD750 and NKA-9, indicating that the adsorption capacity is also related to pore size. The pore size of NKA-9 could facilitate the entry of polyphenol molecules to macroporous resins, but HPD750 could not. In conclusion, the adsorption efficiency of resin might be related to their suitable surface structure, high specific surface area and suitable pore size. Therefore, the adsorption of polyphenols by macroporous resins is possibly mainly due to the molecular sieve formed by the porous network structure and the high specific surface area rather than the Van der Waals force or hydrogen bonding between adsorbent and polyphenols. For desorption, AB-8 and D101 showed the highest efficiency, which might be contributed to the large specific surface area. In the comprehensive consideration of adsorption and desorption rate, D101 was selected as the optimal resin for distiller’s grain polyphenol purification. Zhang et al. also screened macroporous resins to purify flavonoids in *Houttuynia cordatu* Thunb. and demonstrated that D101 was the best [18].

**Figure 1:**
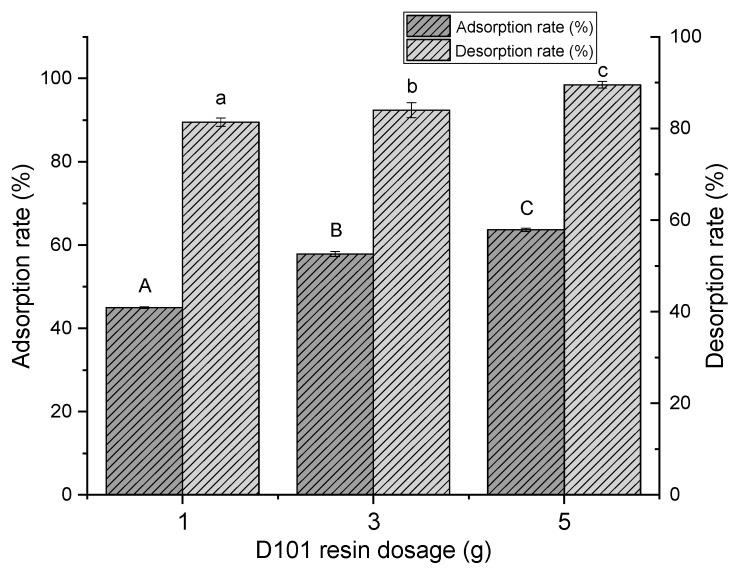
Adsorption and desorption rates of polyphenols from distiller’s grain with different amounts of added D101 resin (1, 3 and 5 g) under the condition of 30 °C and 120 rpm for 24 h. Values with different letters (a–c and A–C) differ significantly (*P* < 0.05) by Duncan’s multiple range test. Vertical bars represent the standard deviation for each value.

A suitable amount of added resin is important in purification, so that the best efficiency can be achieved with the lowest cost. Figure 1 shows the results of the adsorption and desorption rates of D101 at different dosages (1, 3 and 5 g). With the increase in resin dosage, both the adsorption and desorption rates increased remarkably, which was more significant at 1–3 g than 3–5 g. In overall consideration, 3 g of D101 resin was used in follow-up tests.

#### 2.1.2. Static Adsorption Kinetic Curve

**Figure 2:**
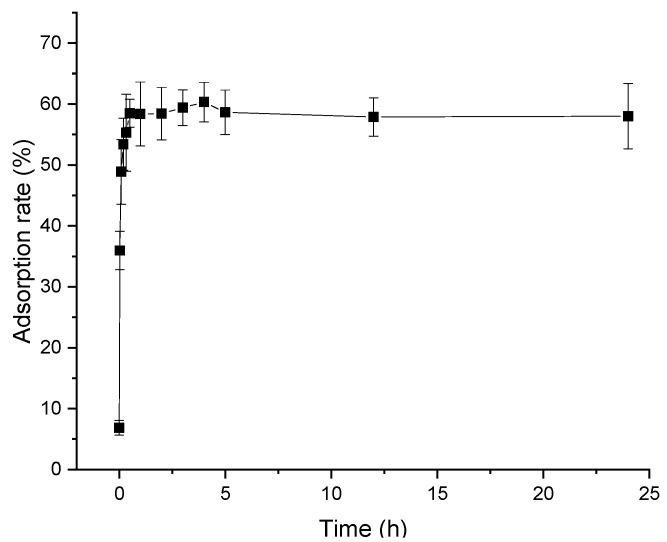
Static adsorption kinetic curve of polyphenols from distiller’s grain with 3 g D101 resin under 30 °C at 120 rpm for 24 h. Vertical bars represent the standard deviation of each value.

Figure 2 demonstrates the static adsorption kinetic curve of polyphenol from distiller’s grain with D101 resin. This figure shows that adsorption rate increased with time until a plateau was reached. During the first stage, a sharply increase in adsorption rate was observed. Afterwards, it increased slowly and tended to balance at four hours, where the adsorption rate was as high as 60.3%. After this, no increase in adsorption rate was observed with time.

The fast initial adsorption rate was possibly due to the occurrence of adsorption in the easily accessible mesopores of the particles. The later slower uptake is indicative of a process with high mass transfer resistance inside the particle [18]. Accordingly, the optimal adsorption time was four hours.

#### 2.1.3. Static Desorption Kinetic Curve

**Figure 3:**
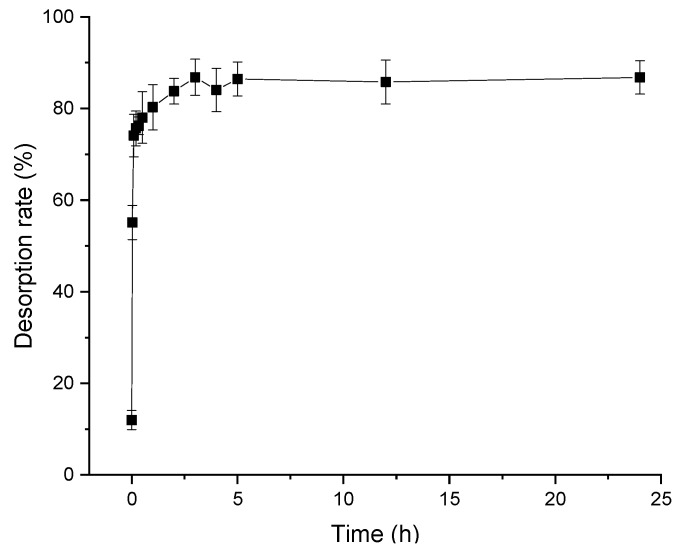
Static desorption kinetic curve of D101 resin (3 g) adsorbing polyphenols from distiller’s grains with 60% ethanol as eluent under the condition of 30 °C at 120 rpm for 24 h. Vertical bars represent the standard deviation of each value.

The static desorption kinetic curve of polyphenol from distiller’s grain with D101 resin is shown in Figure 3. Desorption behavior was parabolic, which could be described as a fast stage followed by a slow desorption, which achieved equilibrium at three hours (desorption rate reached 86%). Thereafter, the desorption rate did not increase with time. As a result, the optimal desorption time was three hours.

#### 2.1.4. Effect of pH and Temperature on Adsorption Efficiency

**Figure 4:**
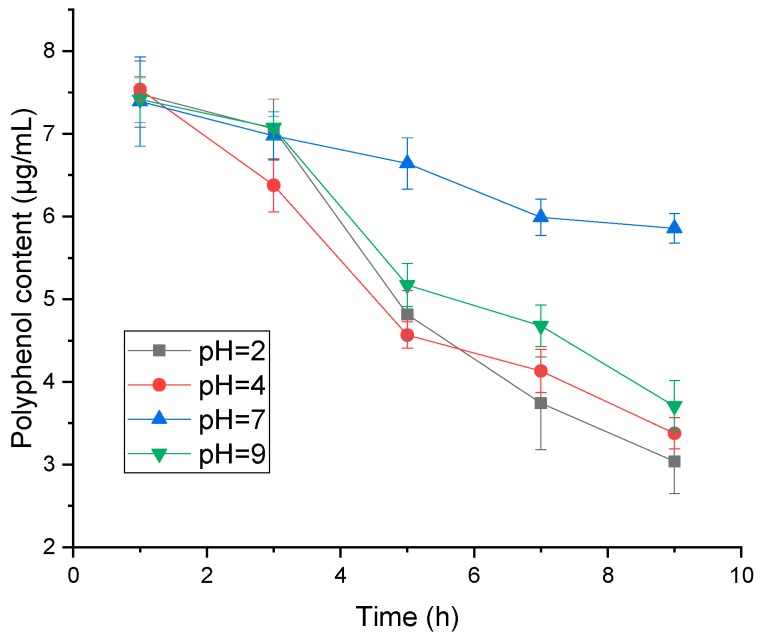
The stability of polyphenol from distiller’s grain in different environments with pH of 2, 4, 7, and 9 at room temperature in dark.

**Figure 5:**
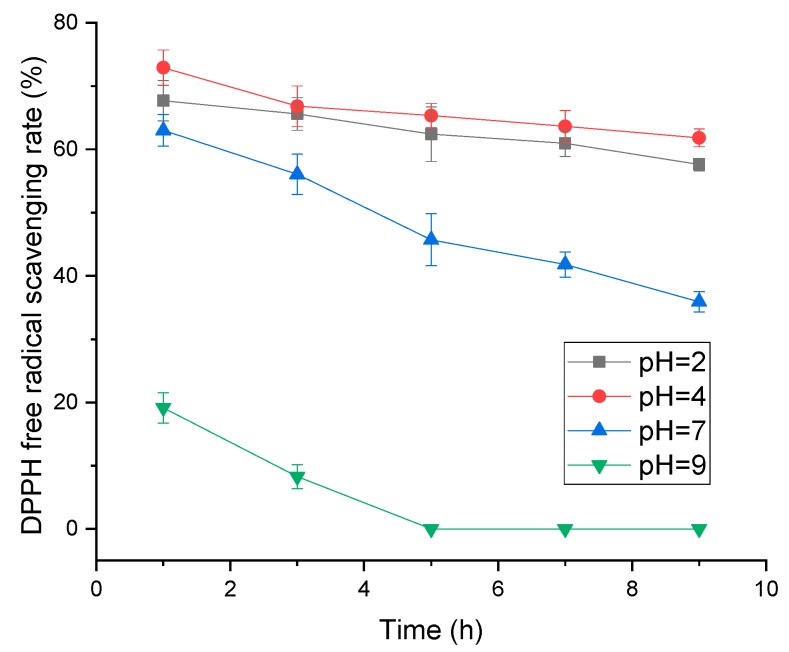
The antioxidant activity of polyphenol from distiller’s grain in different environments with pH of 2, 4, 7, and 9 at room temperature in dark.

**Figure 6:**
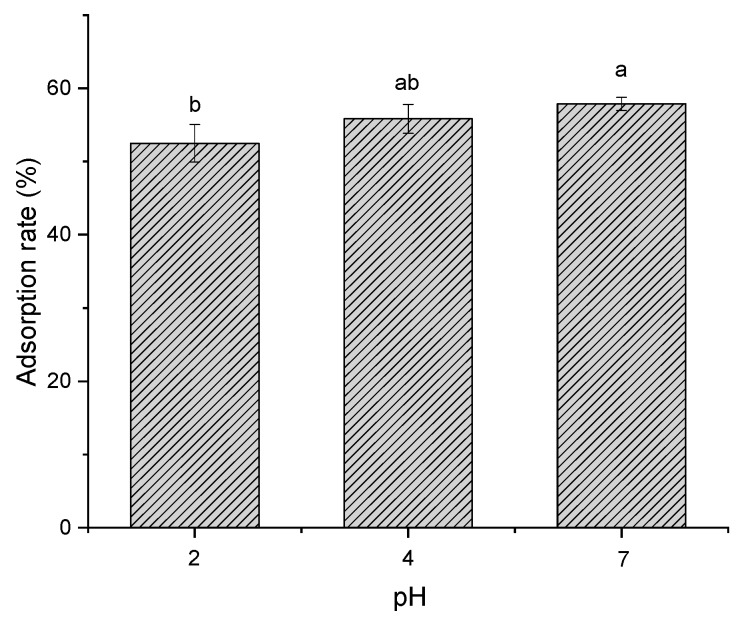
Adsorption efficiency of polyphenols from distiller’s grain with D101 resin (3 g) under the condition of 30 °C at 120 rpm for 24 h in an environment with different pH values of 2, 4, 7 and 9. Values with different letters (a, b) differ significantly (*P* < 0.05) by Duncan’s multiple range test. Vertical bars represent the standard deviation of each value.

Considering that the stability and activity of polyphenols are susceptible to pH value, we have detected polyphenol content, the free radical scavenging rate of DPPH and adsorption rate of polyphenols with resin in different pH environments. As shown in Figure 4, the polyphenol content decreased with time, and the highest one was observed at pH = 7. However, we found that the highest antioxidant activity presented at pH = 4, whereas the lowest one was observed at pH = 9 (Figure 5). It can be explained that some unstable substances may have existed in the polyphenols of distiller’s grains, which could have been easily affected by the alkaline condition. Although pH = 7 exhibited the highest polyphenol content, the antioxidant activity was remarkably lower than pH = 4. In combination with the results of polyphenol stability and antioxidant activity in different pH environments, the effect of pH on adsorption efficiency of polyphenol with resin was conducted at pH = 2, 4 and 7, whereas pH = 9 was excluded due to the observed lowest activity of polyphenols. As shown in Figure 6, we observed no considerable difference in the amount of polyphenol adsorbed onto the adsorbent resin D101 between pH = 4 and 7 environments. Samah et al. have tested the effect of pH (ranging from 3 to 7) on the adsorption of vanillin with resin H103, and noted that pH did not have a significant influence [19]. In the present study, the neutral environment could keep the highest polyphenol content, but the activity was lower than pH = 4. In comprehensive consideration, the optimal environment for purification is pH = 4.

**Figure 7:**
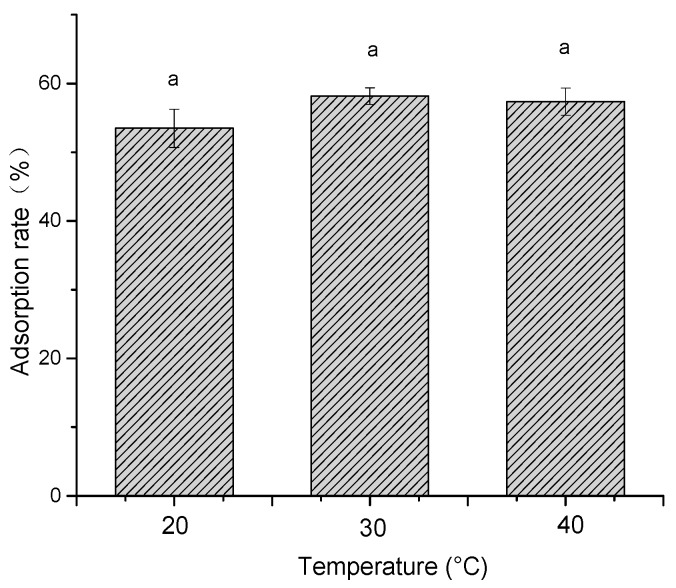
Adsorption efficiency of polyphenols from distiller’s grain using D101 resin (3 g) at different temperatures of 20 °C, 30 °C, and 40 °C under 120 rpm for 24 h. Values with the same letter a differ insignificantly (*P* < 0.05) per Duncan’s multiple range test. Vertical bars represent the standard deviation of each value.

The adsorption efficiency of polyphenols from distiller’s grain with D101 resin at different temperatures of 20, 30 and 40 °C were evaluated. As shown in Figure 7, temperature did not have any obvious influence on polyphenol adsorption using D101 resin. This finding is in accordance with the observation of Samah et al., who reported that temperature did not have any major effect on vanillin adsorption using resin. This result simplifies the subsequent recovery activities, where the separation of polyphenols could be carried out instantly without having to adjust the temperature to any desired level [19].

#### 2.1.5. Effect of Ethanol Volume Fraction on Desorption Efficiency

**Figure 8:**
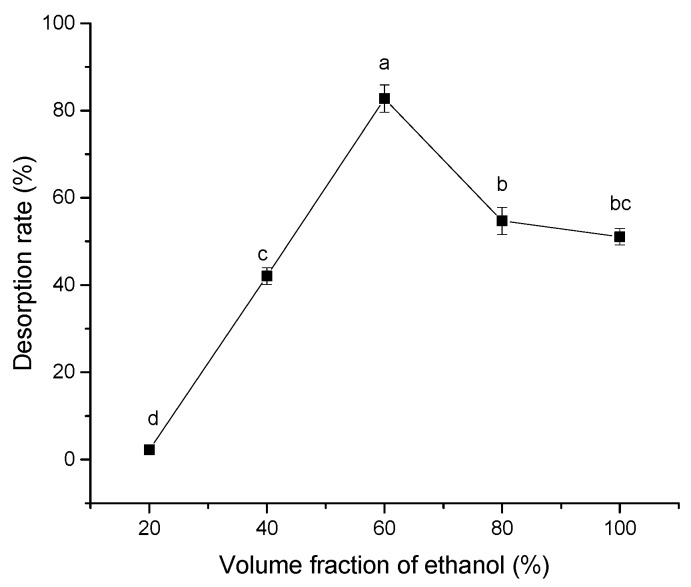
Desorption rate of D101 resin (3 g) adsorbing polyphenols from distiller’s grains with eluent of 20%, 40%, 60%, 80% and 100% ethanol under the condition of 30 °C at 120 rpm for 24 h. Values with the same letter of a differ insignificantly (*P* < 0.05) per Duncan’s multiple range test. Vertical bars represent the standard deviation of each value.

Generally, acetone, methanol, ethanol and other polar solvents have higher desorption rates for polyphenols. However, as polyphenols in distiller’s grains are mainly used in food and pharmaceutical industries, ethanol, which is cheap, easily available, safe, non-toxic, and easy to remove from the solution and recycled, was used as a desorption agent [20,21]. As shown in Figure 8, the concentration of ethanol also affects elution efficiency.

With the increase in the ethanol volume fraction, desorption rate increased and reached a maximum value of 82.7% with a 60% ethanol solution. Afterwards, a downward trend was observed with the increase in ethanol volume fraction. According to Chang et al., desorption of polyphenols from resin is the result of competing interactions between the intermolecular forces of adsorption on the macroporous resin and dissolution in the solvent. When intermolecular forces are recessive, polyphenols desorb from the resin into the solvent [20]. The highest desorption ratio achieved by a 60% ethanol solution might be attributed to the best matching of polarity between the desorption solvent and the adsorbate, thus facilitating desorption.

### 2.2. Antioxidant Activity Comparison

**Figure 9:**
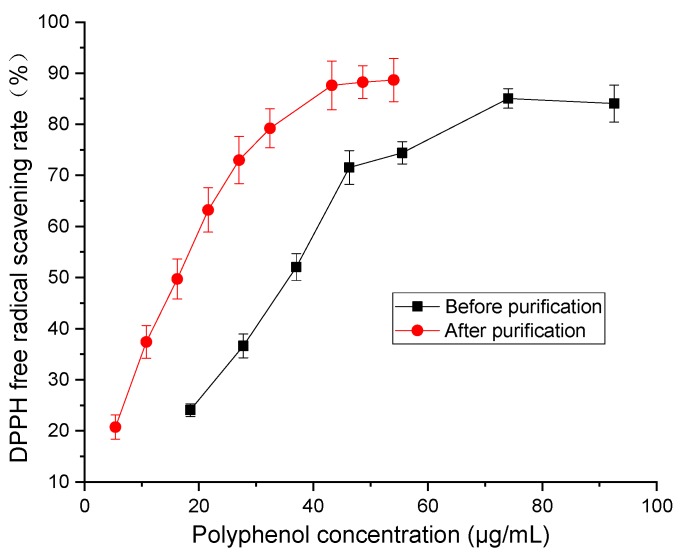
DPPH free radical scavenging rate of polyphenol from distiller’s grain before and after purification with D101 resin under the condition of 30 °C at 120 rpm for 24 h and eluted with 60% ethanol. Vertical bars represent the standard deviation for each value.

To evaluate the purification efficiency of D101 resin, the polyphenol content was detected. The result indicated that the polyphenol concentration was 6.8 and 3.5 mg/g before and after purification, respectively. We calculated that the purification rate was 51%. Additionally, the free radical scavenging rate of DPPH and ABTS was determined to assess the antioxidant activity of polyphenols from distiller’s grain before and after purification. As shown in Figure 9, with the increase in polyphenol concentration, the scavenging rate of DPPH radical increased. At the same concentration of polyphenol, the scavenging capacity of DPPH of polyphenol after purification was stronger than that of samples before purification, with an IC_50_ scavenging capacity for DPPH free radicals of 34.03 and 16.21 μg/mL, respectively. This demonstrated that the antioxidant activity of purified polyphenols is higher than that of unpurified ones, which was attributed to the purification and enrichment of distiller’s grain polyphenols with D101 resin.

**Figure 10:**
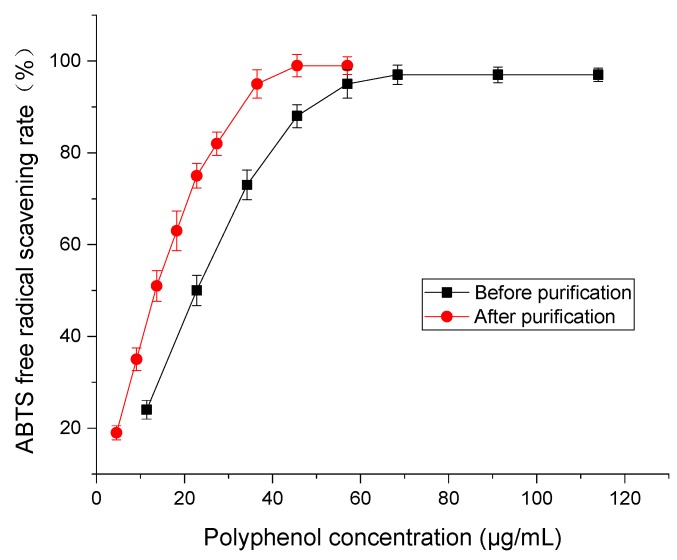
ABTS free radical scavenging rate of polyphenol from distiller’s grain before and after purification by D101 resin under the condition of 30 °C at 120 rpm for 24 h and eluted with 60% ethanol. Vertical bars represent the standard deviation of each value.

ABTS solution is blue-green and highly stable. For antioxidants, where hydrogen supply is available, ABTS transitions to a colorless solution. The antioxidant activity of samples can be calculated according to the change in the ABTS free radical solution absorbance [22]. Figure 10 displays the ABTS free radical scavenging rate of polyphenol from distiller’s grain before and after purification. With the increase in polyphenol concentration, the scavenging capacity of ABTS free radical increased first and then balanced. In comparison, the scavenging rate of polyphenol before purification was higher that of samples after purification. The IC_50_ scavenging capacities of distiller’s grain polyphenols to ABTS free radicals were 20.31 and 5.73 μg/mL, respectively. This shows that purification by D101 resin could improve the antioxidant activity of distiller’s grain polyphenols.

### 2.3. Identification of Phenolic Compounds in Distiller’s Grain Extracts

Phenolic compounds in extracts of distiller’s grain were identified by ultra-performance liquid chromatography tandem mass spectrometry (UPLC-MS/MS) under negative ion scanning mode. Table 2 summarizes the list of compounds and their main fragments observed during UPLC-MS/MS analyses. The typical peaks were first deduced from the literature and then identified by comparing MS data with those of the reference standards. Peak 1 showed a [M − H]^−^ ion peak at *m*/*z* 137, with a characteristic fragment at *m*/*z* 93, which was formed by the loss of CO_2_. The MS fragmentation pattern of peak 1 was in agreement with that of *p*-hydroxybenzoic acid as previous report by Zhao et al., who reported that the parent ion and fragment of *p*-hydroxybenzoic acid were *m*/*z* 137 and 93, respectively [23]. We compared peak 1 to the mass spectra of the authentic standard *p*-hydroxybenzoic acid and the match confirmed that peak 1 was *p*-hydroxybenzoic acid. The molecular ion of peak 2 was observed at *m*/*z* 167 and the characteristic fragment ions were found at *m*/*z* 151.9, 122.9, 107.9 and 91 in the MS^2^ spectrum. Herein, fragment ions of *m*/*z* 151.9 and 122.9 resulted from the elimination of -CH_3_ and CO_2_ from the molecular ion, whereas *m*/*z* 107.9 and 91 suggested the loss of CO_2_/-CH_3_ and O_2_/CO_2_, respectively. As previously reported, peak 2 displayed an identical fragmentation pattern to vanillic acid [24,25]. These fragment ions were in accordance with those of the authentic reference standard of vanillic acid and accordingly, peak 2 was identified as vanillic acid. The parent ion of peak 3 was found at m/z 169, with MS^2^ fragment ions at *m*/*z* 125, indicating a loss of CO_2_. Song et al. and Sawant et al. noted a similar result, indicating that the transition of gallic acid was achieved by MS from *m*/*z* 169 to 125 [26,27]. In combination with the spectra information of the authentic standard of gallic acid, we deduced that peak 3 was gallic acid. Concerning to peak 4, the only MS^2^ fragment ion was seen at *m*/*z* 135, suggesting a loss of CO_2_ from parent ion *m*/*z* 179. Based on the report of Bazylko et al., caffeic acid showed [M − H]^−^ at *m*/*z* 179 and MS^2^ fragment at *m*/*z* 135 [28]. The fragment pattern of peak 4 matched that of the authenticated standard of caffeic acid, suggesting the presence of caffeic acid in distiller’s grain extract. A [M − H]^−^ ion of peak 5 was observed at *m*/*z* 193.1, with MS^2^ fragment ions at *m*/*z* 134, which correspond to the loss of one CO_2_ molecule and -CH_3_ from the parent ion, respectively. According to Karl et al., ferulic acid demonstrated [M − H]^−^ at *m*/*z* 193 and MS^2^ fragment at *m*/*z* 134 [29]. The molecular ion and MS^2^ fragment ions were identical to those of ferulic acid standards, so peak 5 was identified as ferulic acid. Similarly, peak 6 was found to have four fragments at *m*/*z* 182, 152.9, 137.9 and 121, respectively produced by removing -CH_3_, CO_2_, -CH_3_/CO_2_ and CO_2_/O_2_ from [M − H]^−^ *m*/*z* 197. The molecular ion of peak 7 was observed at *m*/*z* 289.1, with the characteristic fragment ions at *m*/*z* 108.7 in the MS^2^ spectrum. Herein, *m*/*z* 108.7 was generated by losing the C_6_H_12_O_6_ parent ion. This spectrum information coincides with that of (−)-epicatechin reported by Wang et al. [30]. In addition to the coincidence with the [M − H]^−^ ion and the MS^2^ fragments of standards of (−)-epicatechin, we inferred that peak 7 was (−)-epicatechin. In terms of peak 8, the [M − H]^−^ ion was *m*/*z* 301.2, which yielded fragment ion *m*/*z* 255.2 by eliminating a H_2_O and CO from *m*/*z* 301.2 [31]. In conclusion, eight phenolic compounds were identified in the extract from distiller’s grains.

### 2.4. UPLC/MS Quantification of Identified Phenolic Compounds in Extracts of Distiller’s Grain

**Figure 11:**
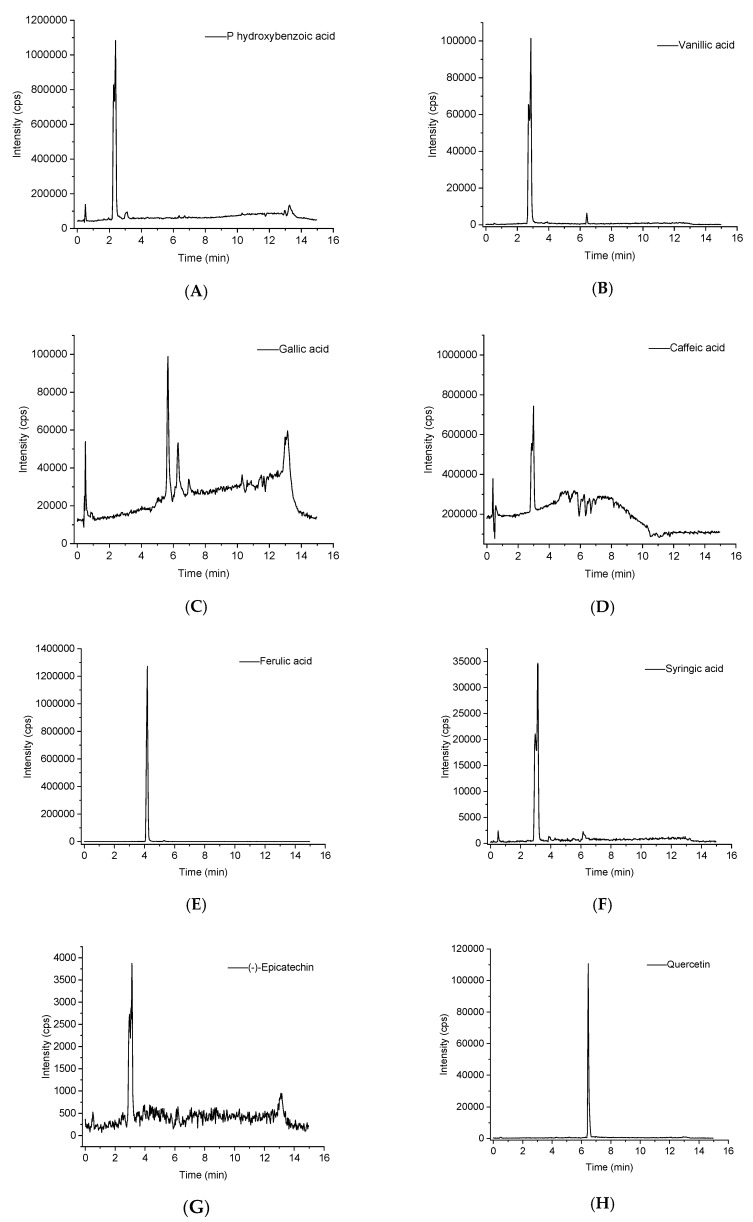
UPLC-MS multiple reaction monitoring (MRM) ion chromatograms of the eight phenolic compounds analyzed: (**A**) p-hydroxybenzoic acid, (**B**) vanillic acid, (**C**) gallic acid, (**D**) caffeic acid, (**E**) ferulic acid, (**F**) syringic acid, (**G**) (−)-epicatechin, (**H**) quercetin.

Based on identification, authenticate standards were applied to quantify the identified compounds in the distiller’s grain extract. Multiple reaction monitoring ion chromatograms of each polyphenol are shown in Figure 11. According to standard curves of the eight standards and peak area of the samples, the concentrations of the identified phenolic compounds were measured. According to Table 3, (−)-epicatechin was the major compound found in distiller’s grain extract which was quantified as 562.7 μg/g extract, followed by ferulic acid (518.2 μg/g), *p*-hydroxybenzoic acid (417.7 μg/g), caffeic acid (217.1 μg/g), syringic acid (158.0 μg/g) and quercetin (147.8 μg/g). Two compounds vanillic acid (66.5 μg/g) and gallic acid (41.4 μg/g) were found in lower concentrations. Based on the above results, we concluded that distiller’s grains contain a variety of phenolic compounds.

In the present study, most of the identified polyphenols can be found either in distiller’s grains or in cereals. Luthria et al. investigated the individual phenolic acids and antioxidant capacity in distillers dried grains formed from corn. They found that corn and distillers grains have a similar phenolic acid composition. The five main phenolic acids were vanillic, caffeic, *p*-coumaric, ferulic, and sinapic acids. Ferulic and *p*-coumaric acids accounted for about 80% of the total identified and quantified phenolic acids [32]. In 2012, Manuela et al. extracted polyphenols from brewer’s spent grain, noting that ferulic acid was the mainly component. They also indicated that other hydroxycinnamic acids and several ferulic acid dehydrodimers, as well as one dehydrotrimer were also present [33]. Lempereur et al. detected ferulic acid in different wheat cultivars and agronomic conditions and found that ferulic acid contents were 0.784 mg/g–7.98 mg/g [34]. These three groups all found that ferulic acid is rich in the by-products of cereal fermentation, which is consistent with our research results. Manach et al. also reported that ferulic acid is the most abundant phenolic acid found in cereal grains [35]. According to Shao and Bao, white rice contains some kinds of polyphenols, with ferulic acid as being the most abundant [36]. Kaur et al. studied polyphenols in millet, showing that different millets have various polyphenol compositions. In all the tested samples, gallic acid, *p*-hydroxybenzoic acid, and caffeic acid were observed, and most samples also contained vanillic acid and syringic acid [37]. The greater diversity of polyphenols found in the present study might be due to the complex composition of the raw fermentation materials of our distiller’s grains.

However, some differences exist between the polyphenol compositions from distiller’s grain extract and the individual cereals, which could be attributed to the influence of industrial food processing on polyphenol content. For example, bolting of cereals can result in a loss of some polyphenols. Grinding of plant tissues may lead to oxidative degradation of polyphenols [35]. Besides, the fermentation of cereals could change the polyphenol composition. Aikpokpodion and Dongo found that the polyphenol content of the fermented cocoa beans dropped from 16.11% on day 0 to 6.01% on day 6 [38]. However, Gan et al. observed different phenomena in the fermentation of eight common edible legumes. They demonstrated that fermentation in general enhanced total phenolic content in all the selected legumes, which could be associated with the biotransformation between soluble phenolics and the release of bound phenolics induced by micro-organisms involved in the fermentation process [39].

## 3. Materials and Methods

### 3.1. Materials

Distiller’s grain, the by-product of liquor production by fermentation of mixture of rice, millet, corn, wheat and sorghum, was a kind gift from Bailaoquan Wine Industry Co., Ltd. (Xinxiang, China). *p*-Hydroxybenzoic acid, vanillic acid, gallic acid, caffeic acid, ferulic acid, syringic acid, (−)-epicatechin, quercetin, 2,2-diphenyl-1-picrylhydrazyl (DPPH) and 2,20-azino-bis(3-ethyl-benzthiazoline-6-sulfonic acid) diammonium salt (ABTS) were purchased from Shanghai Aladdin Biochemical Polytron Technologies Inc. (Shanghai, China). All other chemicals were provided by a local company.

### 3.2. Extraction Procedure

The extraction procedure was performed according to our previous study. Specifically, distiller’s grain was dried at room temperature and then grind by a grinder (ZK-300A, Qingdao Jingcheng Medical Equipment & Pharmaceutical Co., Ltd., Qingdao, China). After 60 mesh screening, 2 g of the power was added into flask and immersed in 40 mL of 60% ethanol. Afterwards, the mixture was treated with ultrasonic at 420 W for 30 min (KQ-700DE, Kunshan Ultrasonic Instruments Co., Ltd., Kunshan, China). After extraction, liquid extracts were separated from solids by centrifugation at 5000 rpm for 10 min (HC-3018R, Anhui USTC Zonkia Scientific Instrument Co., Ltd., Anhui, China). The extraction process was conducted twice. Then the polyphenol concentration was detected with the Folin-Ciocalteu phenol reagent method according to Wang et al. with some modifications [40]. An aliquot (0.8 mL) of the extracted solution was added into brown volumetric bottle with 2.5 mL Folin-Ciocalteu reagent and 4 mL Na_2_CO_3_ (10%). After setting the volume to the scale with distilled water, the mixture was mixed evenly and placed at room temperature for 80 min. Subsequently, the absorbance of the solution was detected at room temperature with a spectrophotometer (UV-752, Shanghai Jinghua Technology Instrument Co., Ltd., Shanghai, China) at 760 nm. Gallic acid was used as standard and results are expressed as mg of gallic acid equivalent (GAE) per mL of distiller’s grain extract.

### 3.3. Purification of Distiller’s Grain Polyphenols

#### 3.3.1. Screening of Macroporous Resin Type and Added Amount

In this experiment, six types of macroporous resins with different polarities, specific surface areas and average pore sizes were selected to purify polyphenols from the distiller’s grain extract. The information about resins is shown in Table 1. Before use, the resins were pretreated by soaking in ethanol for 24 h. After removal of ethanol, the resins were washed with distilled water twice and subsequently treated with 1 N HCl and NaOH solutions successively to remove monomers and porogenic agents trapped inside the pores during the synthesis process. Afterwards, resins were dried at 60 °C under reduced pressure [21].

After activation, 1, 3 and 5 g of resins were added into 20 mL concentrated distiller’s grain extract and the mixture was kept in a shaker (HZQ-F160, Jiangsu Taicang Experimental Equipment Factory, Jiangsu, China) at 30 °C and 120 rpm for 24 h. After adsorption, the supernatant was applied to detect polyphenol content and resins carrying polyphenols were collected for desorption. We added 50 mL of 60% ethanol into the flask to immerse resins, which were incubated at 30 °C and 120 rpm. Twenty-four hours later, the polyphenol level of supernatant was determined. Adsorption and desorption rate were calculated according to Equations (1) and (2). The optimal resin was selected to assay the suitable added amount:

(1) $$\text{Adsorption rate}=(1-\frac{{\mathrm{PC}}_{2}}{{\mathrm{PC}}_{1}})\times 100\%$$

(2) $$\text{Desorption rate}=(1-\frac{{\mathrm{PC}}_{3}}{{\mathrm{PC}}_{1}-{\mathrm{PC}}_{2}})\times 100\%$$

where: PC_1_ is the polyphenol concentration in distiller’s grain extract before adsorption, PC_2_ is the polyphenol concentration in distiller’s grain extract after adsorption, PC_3_ is the polyphenol concentration in the supernatant after desorption.

#### 3.3.2. Static Adsorption Kinetic Curve

Resin D101 (3 g) was added into 20 mL of the concentrated distiller’s grain extract and incubated at 30 °C and 120 rpm. Sampling was conducted at different time points (10, 30, 60, 120, 180, 240, 300, 360, 420, 480, 720 and 1440 min) to detect the polyphenol content.

#### 3.3.3. Static Desorption Kinetic Curve

After adsorption, D101 resins were collected and the surface water was dried with filter paper. Subsequently, they were immersed in 50 mL 60% ethanol and incubated at 30 °C and 120 rpm. Sampling was conducted at different time points (10, 30, 60, 120, 180, 240, 300, 360, 420, 480, 720 and 1440 min) to detect the polyphenol content.

#### 3.3.4. Effect of pH on Polyphenol Stability, Activity and Resin Adsorption Efficiency

The stability and antioxidant activity of polyphenols in different pH environment was investigated. Distiller’s grain extract solution was diluted with acidic and alkaline solutions to obtained pH of 2, 4, 7 and 9. The mixture were placed in dark at room temperature for several hours. Samples were taken out to detect the polyphenol content and DPPH free radical scavenging rate every two hours from the first hour. To evaluate the influence of pH on the adsorption efficiency of the resin, ethanol was used as solvents with the necessary amounts of HCl or NaOH to regulate the liquid pH to 2, 4, 7, and 9. The adsorption process was performed at 30 °C and 120 rpm for 24 h. The adsorption efficiency was calculated according to the polyphenols content and Equation (1).

#### 3.3.5. Effect of Temperature on Adsorption Efficiency

The effect of temperature on adsorption efficiency was also explored by carrying out adsorption process at 20, 30and 40 °C, respectively. Resin D101 (3 g) was added into 20 mL concentrated distiller’s grain extract and incubated at different temperature, 120 rpm for 24 h. The adsorption efficiency was calculated according to the content of polyphenols and Equation (1).

#### 3.3.6. Effect of Eluent Concentration on Desorption Efficiency

Ethanol/water mixtures at different ratios (20%, 40%, 60%, 80% and 100%) were used as solvents to determine the influence of eluent on desorption efficiency. D101 resins adsorbing polyphenols were placed in different ethanol solutions and shaken at 30 °C and 120 rpm for 24 h. Desorption efficiency was calculated according to polyphenol content and Equation (2).

### 3.4. Antioxidant Activity Detection

#### 3.4.1. Free Radical Scavenging Rate of DPPH Detection

DPPH radical scavenging activity of distiller’s grain extracts before and after purification was tested according to the procedure described by Nagai et al. with some modifications [41]. To ensure adequate reaction of the polyphenol samples, a pretest was performed to determine a suitable dosage of samples and DPPH. An aliquot of test sample was gradually added into 2 mL of DPPH radical solution (dissolved in MeOH) until the solution turned colorless. The volume of consumed sample was set as the maximum added amount. Subsequently, we added different amount (20, 40, 80, 120, 160 and 200 μL) of samples and absolute ethyl alcohol into 2 mL of DPPH solution to create a total mixture volume of 3 mL. After mixing, the mixture was placed in the dark for 30 min. Then the absorbance was recorded at 517 nm using a spectrophotometer (UV-752, Shanghai Jinghua Technology Instrument Co., Ltd., Shanghai, China). DPPH solution was set as the control. DPPH radical scavenging activity was calculated using Equation (3):

(3) $$\%\text{DPPH radical scavenging}=\left(1-\frac{A_{X}-A_{0}}{A_{0}}\right)\times 100\%$$

where A_0_ = absorbance of the control and A_X_ = absorbance of the test sample.

#### 3.4.2. Free Radical Scavenging Rate of ABTS Detection

ABTS radical scavenging capacity of extracts was measured by using the method of Ouattara with some modifications [42]. In specific, ABTS powder was dissolved in distilled water to make 7 mM solution and then mixed with 2.45 mM of potassium persulfate solution. After reacted in dark at room temperature for 16 h, the mixture was diluted with ethanol to give an absorbance of 0.7 ± 0.02 units at 734 nm using spectrophotometer. Subsequently, added different amount (80, 160, 240, 320, 400, 480, 640 and 800 μL) of samples and absolute ethyl alcohol into 3.2 mL of ABTS work solution to make the total volume of mixture 4 mL. Afterwards, the mixture was mixed thoroughly and put down in the dark at room temperature for 6 min. Then the absorbance reading was taken by using the spectrophotometer. ABTS radical scavenging capacity was calculated using Equation (4):

(4) $$\%\text{ABTS radical scavenging capacity}=\left(1-\frac{A_{X}{-A}_{0}}{A_{0}}\right)\times 100\%$$

where A_0_ = absorbance of the control and A_X_ = absorbance of the test sample.

### 3.5. Qualitative and Quantitative Analysis of Composition of Distiller’s Grain Polyphenols

The composition of distiller’s grain extract was analyzed by UPLC-MS/MS. Analysis was performed by employing an ACQUITY Ultra Performance LCTM system (1290 UPLCTM, Agilent Technologies Co., Ltd., Beijing, China) with binary solvent manager and single quadrupole micromass ZQ Mass Detector (Triple QuardTM 5500, AB SCIEX Pte Ltd., Beijing, China) coupled with an electrospray ion source operating in negative mode.

To detect the content of the identified polyphenolic compounds, standards were used and detection was conducted using UPLC-MS. The MS signal was used only for qualitative analysis based on specific mass spectra of each polyphenol. Quantification was conducted using the area under the mass spectral peak for individual multiple reaction monitoring ion channels for each polyphenol and comparing it to a standard curve. For separation, a reversed phase analytical column was used (Zorbax Eclipse Plus C18 2.1 × 50 mm i.d., 1.8 μm) and the working temperature was 30 °C. The UPLC was follows: Solvent A was composed of 65% methanol and 34.5% water containing 0.5% acetic acid and solvent B of water with 0.5% acetic acid. The elution was complete using the following gradient: 0–3.5 min 15% A, 3.5–6.0 min 30–35% A, 6–7.5 min 35–75% A, 7.5–15 min 15% A. The injection volume was 2.5 μL and flow rate was 0.3 mL/min.

### 3.6. Statistical Analysis of Data

To compare treatments, statistical analysis was conducted using SPSS 18.0 and significant differences were verified by one-way ANOVA with Duncan’s multiple range test (*p* < 0.05). All graphs were drawn by using Origin Pro 2015 (OriginLab Corporation, Northampton, MA, USA). All assays were repeated three times.

## 4. Conclusions

In this study, we developed a method of purifying phenolic compounds from distiller’s grain. The phenolic compounds were extracted from distiller’s grain using an ultrasound approach and purification using a macroporous resin, through which the antioxidant activity of the phenolic compounds was increased two to four times. The phenolic compounds in distiller’s grain extract were identified and sorted according to their contents as follows: (−)-epicatechin > ferulic acid > *p*-hydroxy-benzoic acid > caffeic acid > syringic acid > quercetin > vanillic acid > gallic acid. The abundant polyphenol content and purification method provides a new method for the secondary use of distiller’s grains.

## Figures and Tables

**Table 1:** Physical properties and adsorption and desorption rates of six types of macroporous resin.

Resin Type	Polarity	Specific Surface Area/(m^2^/g)	Average Pore Size (Å)	Adsorption Rate (%)	Desorption Rate (%)
AB-8	weakly polar	480–520	130–140	35.56 ± 0.35b	97.39 ± 0.23a
D101	nonpolar	600–700	100–110	44.99 ± 0.11a	98.47 ± 0.19a
HPD400	nonpolar	500–550	75–80	45.71 ± 0.63a	86.94 ± 0.56b
HPD750	middle polar	650–700	85–90	27.00 ± 0.16d	94.19 ± 0.39ab
NAK-9	polar	250–290	155–165	44.29 ± 0.78a	92.46 ± 0.13ab
S-8	polar	100–120	280–300	28.81 ± 0.40c	90.33 ± 0.22ab

Note: Values are mean ± S.D., and different letters within same column indicate *P* ≤ 0.05. Any means in the same column followed by different letters are significantly (*P* < 0.05) different by Duncan’s multiple range test.

**Table 2:** Identification of phenolic compounds from distiller’s grain extract by UPLC-MS/MS.

Peak	[M − H]^−^	Compounds	MS^2^
A1	137	*p*-Hydroxybenzoic acid	93
A2	167	Vanillic acid	151.9, 122.9, 107.9, 91
A3	168.9	Gallic acid	125, 107, 97, 81, 78.9, 69
A4	179	Caffeic acid	135
A5	193.1	Ferulic acid	178.1, 149, 134, 117
A6	197	Syringic acid	182, 152.9, 137.9, 121
A7	289.1	(−)-Epicatechin	202.8, 197.1, 153, 151.1, 121, 108.7
A8	301.2	Quercetin	255.2, 179, 150.8, 121.1, 107

**Table 3:** Identified polyphenol contents of distiller’s grain extract tested by UPLC-MS.

Polyphenols	Retention Time (min)	Concentrations (μg/g Extract)
*p*-Hydroxybenzoic acid	2.39	417.7
Vanillic acid	2.86	66.5
Gallic acid	0.51	41.4
Caffeic acid	2.99	217.1
Ferulic acid	4.19	518.2
Syringic acid	3.14	158.0
(−)-Epicatechin	3.13	562.7
Quercetin	6.46	147.8
